# Simultaneous renal infarction and splenic infarction as a possible initial manifestation of COVID‐19: A case report

**DOI:** 10.1002/ccr3.4819

**Published:** 2021-11-06

**Authors:** Abdulrahman F. Al‐Mashdali, Akram F. Alwarqi, Saffa M. Elawad

**Affiliations:** ^1^ Department of Internal Medicine Hamad Medical Corporation Doha Qatar; ^2^ Department of Radiology Hamad Medical Corporation Doha Qatar; ^3^ Department of Nephrology Hamad Medical Corporation Doha Qatar

**Keywords:** acute kidney injury, COVID‐19, renal infarction, splenic infarction

## Abstract

We recommend testing for SARS‐CoV‐2 in a patient with an unexplained thromboembolic event, even in the absence of fever or respiratory symptoms.

## Graphical abstract

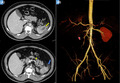

## 1 BACKGROUND

COVID‐19 is an emerging condition associated with various complications. Coagulopathy and thromboembolic events in COVID‐19 might occur even without fever and other classic respiratory symptoms. Rarely, COVID‐19 patients can present initially with acute abdominal pain due to renal or splenic infarctions.

Acute renal infarction is a rare condition that occurs due to the disruption of renal artery supply to the kidneys and is usually caused by thromboembolic diseases.^1^The incidence of renal infarction was estimated to be between 0.004% and 0.007% of cases presenting to the emergency department.^2^ However, in a study of autopsies, renal infarction was found in 1.4% of cases,^3^ which reflects that the incidence of renal infarction may be higher than estimated in the literature, because patients with renal infarction mainly present with abdominal or flank pain that mimics other familiar conditions, such as renal colic and acute pyelonephritis; hence, the diagnosis of renal infarction is more likely to be delayed or missed.^4^

Recently, severe acute respiratory syndrome coronavirus‐2 (SARS‐CoV‐2) infection has been linked to a hypercoagulable state, leading to arterial and venous thrombotic events. Pulmonary embolism has been the most prevalent thrombotic event in the COVID‐19 era.^5^ Renal infarction and splenic infarction associated with COVID‐19 have been described in the few case reports. Coagulopathy in COVID‐19 may be present even in the absence of classic respiratory symptoms and fever.^6^, ^7^ Infrequently, COVID‐19 can present initially with acute abdominal pain due to renal or splenic infarctions.^8^, ^9^

Here, we report a case of simultaneous renal infarction and splenic infarction presenting initially with an acute right flank pain and an acute kidney injury. Our patient was discovered to have a COVID‐19 infection during his hospital admission, and thorough investigations for cardioembolic and thrombophilia causes came negative. Accordingly, we argue that acute renal infarction was the initial presenting manifestation of COVID‐19 in our patient, and COVID‐19 polymerase chain reaction (PCR) should have been done on the first day of presentation.

## 2 CASE PRESENTATION

A 43‐year‐old male patient presented to the emergency department with severe right‐sided flank pain of sudden onset that was associated with nausea and vomiting. He denied fever, dysuria, or change in his bowel habit. Also, he did not report respiratory symptoms or recent contact with sick people. His past medical history was only significant for type B aortic dissection diagnosed 4 months before this presentation and treated medically with a beta‐blocker. Also, he had deafness since childhood of unknown cause. The only medications that he was on before this presentation was bisoprolol 5 mg once daily. He denied any personal or family history of thromboembolic events. He is a smoker, but denied alcohol intake or substance abuse. He had a private business, married, and have children.

**FIGURE 1:**
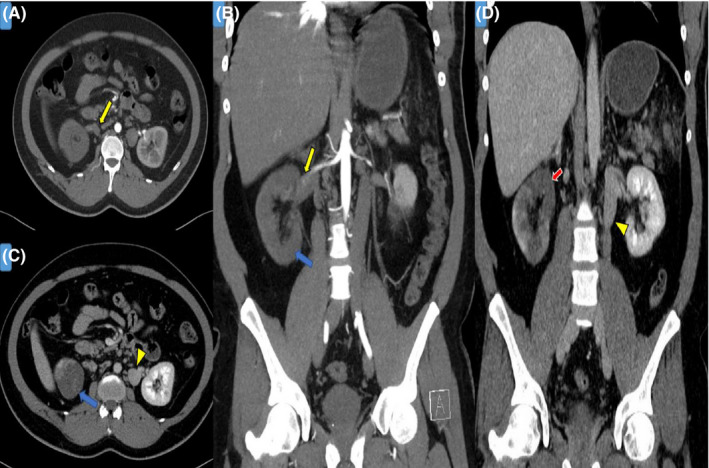
Images from Abdominal CT scan with contrast. (A and B) the arterial phase, and (C and D) the venous phase demonstrate right renal arterial filling defect (yellow arrows). The right kidney parenchyma did not fill with contrast during the arterial phase (blue arrows) comparing to the left kidney and persistent of the right upper kidney hypoattenuation during venous phase suggestive of renal infarction (red arrow)

**FIGURE 2:**
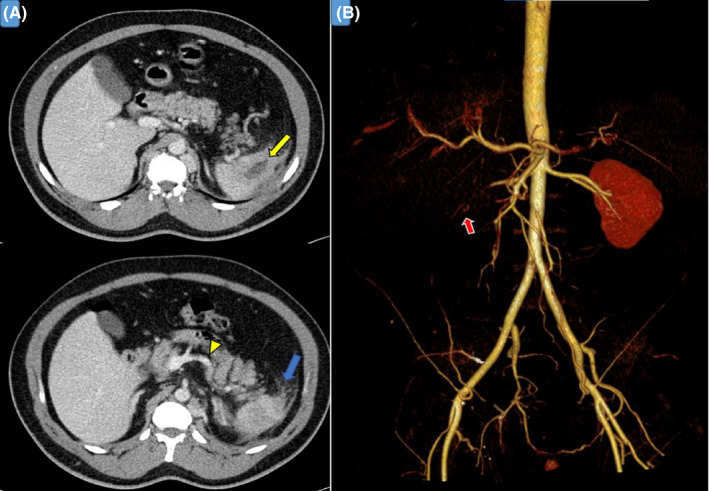
Images from Abdominal CT scan with contrast and 3D reformats. (A) shows splenic hypodense lesion (yellow arrow), which suggests splenic infarct with peri splenic fat stranding (blue arrow) despite normal splenic artery patency (yellow arrowhead). (B) demonstrates the absence of renal perfusion (red arrow) due to nearly total right renal artery occlusion

His vital signs indicated an oral temperature of 36.9°C, blood pressure of 125/80 mmHg, his pulse was regular and within normal range, and he was not tachypneic with an oxygen saturation of 96% on room air. Physical examination revealed tenderness in the right flank region without guarding or rigidity, and apart from that, physical examination was unremarkable. Laboratory abnormalities on presentation included white blood cells of 15.6 × 10^3^/μl, creatinine level of 146 mmol/L (his baseline creatinine, done 1 month before this presentation, was normal), estimated glomerular filtration rate 45 mL/min/1.73 m^2^, D‐dimer 3.17 mg/L (elevated), and C‐reactive protein of 34 mg/L. All relevant laboratory data are summarized in Table 1. Urinalysis revealed proteinuria and hematuria. Accordingly, renal colic was on top of the differential diagnosis. However, the urinary tract's nonenhanced CT ruled out nephrolithiasis and showed multiple hypodensities in the kidneys and spleen. Abdominal CT with contrast was requested to confirm the diagnosis of renal infarction and revealed right kidney infarction and splenic infarction (Figures 1 and 2). Electrocardiography (ECG), echocardiography, and Holter 48‐h monitoring were normal (no evidence of atrial fibrillation or cardioembolic source).

**TABLE 1:** Laboratory findings on admission

Parameter	Value	Reference range
WBC	15.6 × 10^3^/μl	4.0–10.0
Hgb	16.4 gm/dl	13.0–17.0
Platelet	459 × 10^3^/μl	150–400
PT	10.9 s	9–12
PTT	28.2 s	24–32
INR	1.1	0.8–1.2
D‐Dimer	3.17 mg/L	0.00–0.46
Fibrinogen	6.87 gm/L	1.70–4.20
Urea	5.9 mmol/L	2.5–7.8
Creatinine	146 μmol/L	62–106
Sodium	138 mmol/L	133–146
Potassium	4.3 mmol/L	3.5–5.3
ALT	27 U/L	0–41
AST	22 U/L	0–40
Lipase	38 U/L	13–60
Lactate Dehydrogenase	320 U/L	135–214
CRP	34.4 mg/L	0.0–5.0
Procalcitonin	0.03 ng/ml	<0.05
Serum Ferritin	488 μg/L	18–340
Interleukin ‐ 6	13 pg/ml	≤7

Abbreviations: ALT, alanine aminotransferase; APTT, activated partial thromboplastin time; AST, aspartate aminotransferase; CRP, C‐reactive protein; Hgb, hemoglobin; INR, International normalization ratio; PT, prothrombin time; WBC, white blood cell.

The patient was started on heparin infusion, because of AKI, after the agreement between medical, vascular surgery, and nephrology teams. On day 4 of the hospital stay, the patient developed fever and dry cough, and COVID‐19 PCR came positive. The chest x‐ray was unremarkable. COVID‐19 test was not done on admission due to the absence of fever and the other COVID‐related symptoms. Then, the patient was transferred to the COVID‐19 facility. During his hospital stay in the COVID‐19 facility, the patient did not require any oxygen support and managed conservatively till he finished his quarantine period.

Investigations for inherited thrombophilia, antiphospholipid syndrome, and vasculitis were negative (Table 2). On day 12 of the hospital stay, the patient was discharged home with a prescription of warfarin at the dose of 3.5 mg and an INR target of 2–3.

**TABLE 2:** Thrombophilia and vasculitis workup

Parameter	Value	Normal range
JAK2 V617F analysis.	Negative	
Prothrombin c.*97G>A variant	Negative	
Factor V Leiden c.1601G>A variant	Negative	
Protein C activity	83.6%	70.0–140.0
Protein S activity	100.5%	72.0–126.0
Antithrombin activity	83.5%	79.4–112.0
Lupus anticoagulant	Negative	
Anti‐Cardiolipin Ab IgG	Negative	
Anti‐Cardiolipin Ab IgM	Negative	
Anti B2 glycoprotein IgG	Negative	
Anti B2 glycoprotein IgM	Negative	
ANA	Negative	
ANCA	Negative	
C3	1.28 gm/L	0.90–1.80
C4	0.34 gm/L	0.10–0.40

Abbreviations: ANA, antinuclear antibodies; ANCA, antineutrophil cytoplasmic antibodies.

He was planned to be followed in nephrology and warfarin clinics for kidney function and INR monitoring. At 5 months of follow‐up in our outpatient clinic, the patient was asymptomatic (with no recurrent thrombotic event) and vitally stable with INR within the target level. However, his creatinine level was still elevated (139 mmol/L, compared to 146 mmol/L on presentation). Accordingly, the diagnosis of CKD (stage 3) was established, most likely related to his history of renal infarction.

## 3 DISCUSSION

Cardioembolic diseases, renal artery injury, and hypercoagulability states are the three major etiologies of renal infarction.^10^ Atrial fibrillation is considered as the most common etiology for renal infarction, found in more than 60% of the cases.^11^ Renal infarction was also reported on rare occasions in association with other disorders, including but not limited to aortic dissection, vasculitis, systemic lupus erythematosus (SLE), septic emboli due to infective endocarditis, sickle cell diseases, and renal arteries fibromuscular dysplasia (FMD). Nevertheless, the cause of renal infarction could not be identified in around 30% of the reported cases.^12^ Most patients with renal infarction present with abdominal or flank pain; thus, renal infarction may be misdiagnosed initially as acute renal colic or acute pyelonephritis.^1^ In a retrospective study of 14 patients, renal infarction was initially misdiagnosed as renal colic.^13^ Renal infarction diagnosis is often reached after 48 h from the onset of symptoms.^14^In ED, our patient was diagnosed with renal colic based on his clinical presentation, which was excluded by urinary tract's nonenhanced CT.

Renal infarction is associated with elevated lactate dehydrogenase level (LDH) in more than 90% of the cases, which may help in the differentiation of this disease from renal colic and pyelonephritis.^1^ As mentioned above, many patients with renal infarctions can be misdiagnosed as renal colic at the start, and appropriately, nonenhanced CT of the urinary tract, as a gold standard in evaluating ureteric colic, will be offered and might fail to detect renal infarction. Therefore, it is recommended to consider contrast‐enhanced abdominal CT if the urinary stone had been ruled out by the nonenhanced CT, and renal infarction is still highly suspected.^15^

One of the most important diagnostic tools to diagnose renovascular disease is a CT scan with contrast.^16^The vascularity of the kidneys is usually evaluated during the vascular phase, which is around 15–25 s after intravenous contrast administration; after that, corticomedullary differentiation (nephrogenic phase) can be obtained after around 25–80 s after the contrast agent given as well as the appearance of the collecting system in the execratory phase, after approximately 3–5 min.^17^ The vascular manifestations are not common in the CT scan. The kidney's parenchymal appearance depends on the embolus size, location, and age. The Absence of renal tissue enhancement in the arterial phase is a one clue for the diagnosis. A wedge‐shaped area of hypoattenuation in the kidney can be seen, but in the global infarction, the kidney is enlarged, and its reniform configuration can be preserved.^18^

Acute kidney injury (AKI) is the main complication of renal infarction, especially in patients with bilateral renal infarction. AKI was found in 40‐76% of the cases.^19^, ^20^ Typically, AKI due to renal infarction will improve gradually with time; however, some patients occasionally develop chronic kidney diseases (CKD) and even require long‐term dialysis. It was found that higher LDH level is correlated with more extensive kidney infarction and subsequently higher risk of AKI and CKD in those patients.^21^

The pathophysiology of venous thrombosis in COVID‐19 is complex with the involvement of the three components of the Virchow triad (hypercoagulability, vessel wall damage, and blood stasis). In severe COVID‐19 cases, hyperinflammatory reactions stimulate the productions of clotting factors and activate the circulating platelets, creating a hypercoagulable state. On the other hand, it was found that the virus can directly attack the endothelial cells of the vessels, leading to diffuse endotheliitis and the subsequent thrombus formation due to vascular wall damage. In addition, immobility (during hospital admission) plays an essential role in thrombus formation due to blood stasis. Of note, the aforementioned mechanisms were established for COVID‐19 related venous thrombosis; however, the pathophysiology of arterial thrombosis in COVID‐19 patients, such as renal or splenic artery thrombosis, is still not well known.^22^, ^23^, ^24^

Splenic infarction might occur as a result of arterial or venous thrombosis. The most typical presentation includes left upper quadrant abdominal pain, occurring in more than 50% of the cases. However, splenic infarction is sometimes discovered incidentally with abdominal imaging for other reasons. The most common etiologies are thromboembolic diseases (such as atrial fibrillation and infective endocarditis) and hypercoagulable disorders (such as sickle cell disease, polycythemia vera, and malignancy). Management of splenic infarct is often supportive, although some cases might require splenectomy.^25^, ^26^ Since the beginning of the COVID‐19 pandemic, several cases of splenic infarction have been connected to COVID‐19, especially with severe cases.^5^, ^24^ Our patient was also found to have asymptomatic splenic infarction in addition to the main presentation with acute renal infarction.

SARS‐CoV‐2 can affect the kidneys through multiple mechanisms, such as a renal hypoperfusion, a consequence of cytokines storm, and a direct cytotoxic effect on nephrons. Also, renal infarction has been reported in COVID‐19 patients, given the hypercoagulable state associated with this condition.^5^ In our case, COVID‐19 was discovered during the hospital stay, and unfortunately, COVID‐19 screening was not done on the day of admission due to the lack of fever or other classic symptoms. Extensive workup for thrombophilia, antiphospholipid syndrome, and vasculitis was negative in our patient (Table 2), so COVID‐19 is the only possible predisposing factor for renal infarction in our case. Therefore, we wonder whether COVID‐19 was a predisposing factor for renal infarction in our patient or just a coincidence.

## 4 CONCLUSION

Renal infarction and splenic infarction can be easily misdiagnosed because of their nonspecific clinical manifestations, which mimic other common diseases. Accordingly, they should be considered in any patient presenting with unexplained abdominal pain, mainly patients at risk for thromboembolism are known to have a hypercoagulability disorder. Contrast‐enhanced CT is crucial for the diagnosis of both conditions. Of note, Thrombotic complications, such as renal infarction or splenic infarction, might precede the classic manifestations of COVID‐19. Therefore, we suggest testing for SARS‐CoV‐2 in a patient with unexplained renal infarction or splenic infarction, even in the absence of fever or respiratory symptoms.

## CONFLICTS OF INTEREST

The authors have no conflicts of interest to declare.

## AUTHOR CONTRIBUTIONS

AA contributed to data collection, literature review, and manuscript writing.AW contributed to the clinical radiology part. SE contributed to the literature review and final revision of the manuscript as a mentor.

## ETHICAL APPROVAL

This case report was approved by the Hamad Medical Corporation's Medical Research Center (Protocol number: MRC‐04‐21‐288).

## CONSENT

Written informed consent was obtained from the patient for the publication of this case report.

## Data Availability

The datasets used and/or analyzed during the current study are available from the corresponding author on request.
